# A dataset on anthropometric measurements of children in Jordan for ergonomic product design

**DOI:** 10.1016/j.dib.2024.110123

**Published:** 2024-02-01

**Authors:** Mumen Rababah, Morad Etier

**Affiliations:** Department of Industrial Engineering, Faculty of Engineering, The Hashemite University, P.O. Box 330127, Zarqa 13133, Jordan

**Keywords:** Ergonomics, Human factors, Children anthropometry, Product design

## Abstract

The assessment and examination of anthropometric measures in children are of paramount significance in the advancement and creation of furniture, tools, and toys that specifically addresses the requirements of children as users. The dataset analyzed a total of 354 children from Jordan, divided into six distinct age groups ranging from 6 months to 9 years. The linear static measures included the skeletal dimensions pertaining to the distances between joints in the body, as well as the dimensions of the middle and lower bodies. It consists of 23 anthropometric measurements such as: stature, sitting height, knee height, eye height sitting, chest depth and shoulder breadth and others. The dataset supports the article “Preliminary and Comprehensive Static Anthropometric Measurements of Jordanian Children for Various Age Groups” [1]. The available data may initiate the establishment of a connection between anthropometric measurements and other design attributes used in Jordanian society. Additionally, these data have the potential to provide valuable insights for the development of diverse functional products, including clothing and safety equipment specifically tailored for the needs of children in Jordan.

Specifications Table

**Table:** 

Subject	Ergonomics and human engineering
Specific subject area	Anthropometry
Data format	Raw
Type of data	Tables and Figures
Data collection	354 children (6 months – 9 years age range) were considered for the analysis and the anthropometric measurements are 23 measures namely: stature, sitting height, back of the head height, ear height sitting, eye height sitting, height of prominent neck vertebra, shoulder height sitting, chest depth, lower abdominal depth, waist height sitting, hip breadth sitting, underside of elbow height to floor (sitting), underside of elbow height to seat (sitting), back of elbow to wrist crease, shoulder breadth, back of elbow to fingertip, shoulder finger tips depth, palm breadth, hand breadth, wrist breadth, index finger height, middle finger height, seat height, popliteal height (sitting), top of knee height (sitting), lumbar popliteal depth.
Data source location	Cities in Jordan (Amman, Irbid, Zarqa)
Data accessibility	Repository name: Mendeley Data Repository. Data identification number: 10.17632/833pdbrhdr.2 Direct URL to data: https://data.mendeley.com/datasets/833pdbrhdr/2
Related research article	Etier, M., Alkhazali, A., Alsukker, A., Al-Momani, E., Asha, S., & Al-Sharif, A. (2021). Preliminary and Comprehensive Static Anthropometric Measurements of Jordanian Children for Various Age Groups. Ergonomics in Design, 29(3), 11-25. https://doi.org/10.1177/1064804620947002

## 1 Value of the Data

• The advancement and creation of furniture, tools, and games that specifically addresses the requirements of children as users.
• The potential to provide valuable insights for the development of diverse functional products, including clothing and safety equipment specifically tailored for the needs of children in Jordan.
• The aforementioned data has immense value for researchers engaged in the study of child product development, particularly in the area of designing products with a focus on ergonomics.

## 2 Background

Anthropometric measurements are pivotal for enhancing convenience and comfort in designs, especially tailored for children. The establishment of this dataset stems from a deliberate effort to address the current gap in anthropometric data, specifically within pediatric statistics in Jordan. Given the rapid physical growth and maturation characterizing children’s development, a comprehensive understanding of their anthropometric characteristics is essential for designing items that meet their needs and enhance overall usability. The dataset was collected using a meticulous technique that involved collecting anthropometric measurements from a representative sample of Jordanian children.

It is noteworthy that this data article does not make conclusive or interpretive statements; however, its significance comes from its potential to serve as a foundational resource for researchers, designers, and practitioners actively involved in the field of ergonomic product development. By providing detailed anthropometric data, this dataset not only reinforces the related research paper's findings and conclusions, but also broadens its applicability by serving as a valuable resource for future investigations and endeavors in the field of ergonomic product design for children in Jordan.

## 3 Data Description

**Fig. 1:**
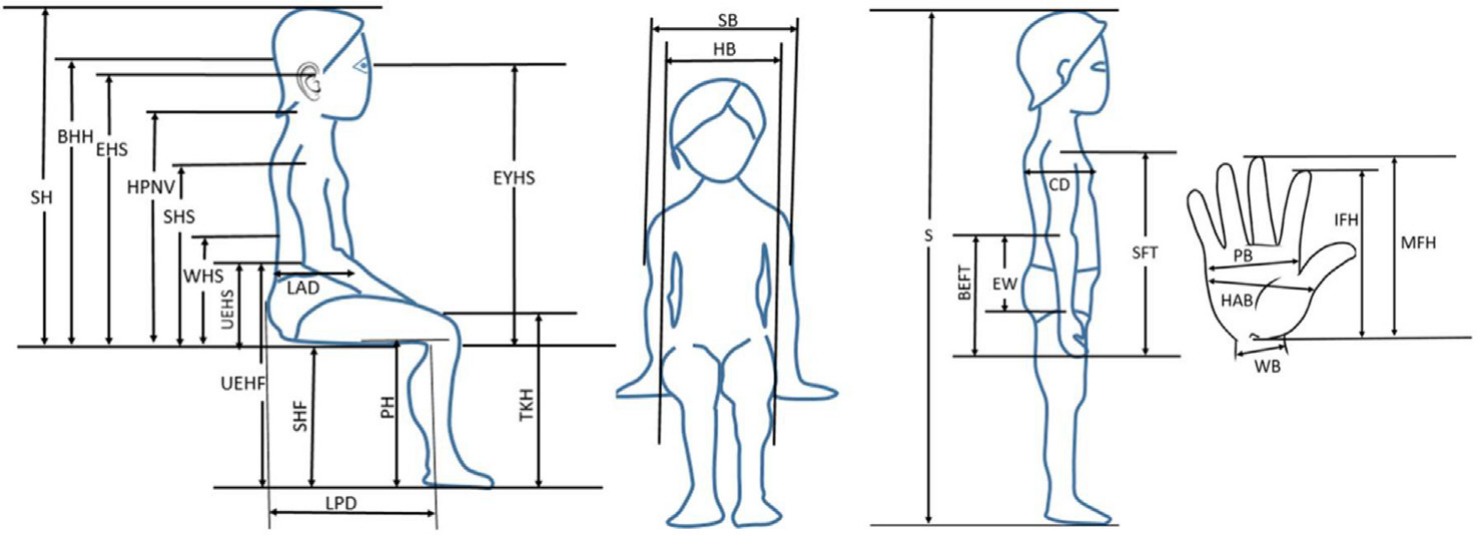
Children's body dimensions [1].

The provided data consists of tables that give anthropometric measurements of children's bodies. Additionally, the available data provides substantial support for a research article focused on the areas of product design, development, and evaluation [1]. Human factors engineers often use anthropometry to enhance daily consumer items and improve the work environment, hence increasing safety and comfort. Anthropometry is an academic discipline that focuses on the study of human body proportions and physical features [2]. Table 1 displays the characteristics of the measuring instruments used in the acquisition of anthropometric data. Table 2 shows the distribution of children who were involved in the experiment. Table 3 and Fig. 1 provide a comprehensive list of 23 unique anthropometric dimensions along with their respective abbreviations and explanations. The all-encompassing dataset, which includes all relevant tables, can be accessed via the Mendeley Data repository, providing users with unrestricted and transparent entry to its contents.

**Table 1:** Characteristics of the measuring devices used in getting the anthropometric data.

Tool	Characteristics
Lafayette Large Caliper	Company: Lafayette Instrument Company Model Number: 01290 Measurement Range: 0–60 cm Accuracy: 0.5 mm
Lafayette Small Caliper	Company: Lafayette Instrument Company Model Number: 01291 Measurement Range: 0–30 cm Accuracy: 0.5 mm
Lafayette Chest Depth	Company: Lafayette Instrument Company Model Number: 01140 Measurement Range: 0–60 cm Accuracy: 0.5 mm
Vernier Caliper	Company: Mitotoyo Model Number: 530/104 Measurement Range: 0–150 mm Accuracy: 0.1 mm
Digital Bathroom Scale	Company: Taylor Precision Products Model Number: 7506 Measurement Range: 0–180 kg Accuracy: 0.05 kg

**Table 2:** Distribution of children by gender, age groups, and location.

Ages	Males	Females	Sum	Percentage	Amman	Irbid	Zarqa
6–12 months	35	35	70	19.77%	70	0	0
13–24 months	20	20	40	11.30%	40	0	0
2–36 months	20	20	40	11.30%	17	21	2
3–5 years	20	20	40	11.30%	18	22	0
5–7 years	35	35	70	19.77%	2	41	27
7–9 years	47	47	94	26.55%	14	40	40
Total	177	177	354	100.00%	161	124	69

**Table 3:** The Anthropometric dimensions’ abbreviations and their meanings.

Abbreviation	Meaning	Illustration
S	Stature	The linear distance from top of the head to the floor when standing
SH	Sitting Height	The linear distance from the top of the head to the seat when sitting
BHH	Back of The Head Height	The distance between the back of the head (the middle of the back head) to the seat when sitting.
EHS	Ear Height Sitting	The linear distance between the middle of the ear to the seat when sitting
EYHS	Eye Height Sitting	The linear distance between the middle of the eye to the seat when sitting.
HPNV	Height of Prominent Neck Vertebra	The distance between the back of the head (first cervical vertebrae) to the seat when sitting.
SHS	Shoulder Height Sitting	The linear distance between the maximum point of the shoulders to the seat when sitting.
CD	Chest Depth	The depth of the chest when standing.
LAD	Lower Abdominal Depth	The depth of the lower abdominal
WHS	Waist Height Sitting	The linear distance between the waist and the seat when sitting
HB	Hip Breadth Sitting	The breadth of hip when sitting
UEHF	Underside of Elbow Height to Floor (Sitting)	The distance between the bottom of the elbow to the floor when sitting
UEHS	Underside of Elbow Height to Seat (Sitting)	The distance between the bottom of the elbow to the seat when sitting.
EW	Back of Elbow to Wrist Crease	The linear distance between the elbow to the beginning of the write when standing
SB	Shoulder Breadth	The breadth of the shoulder when sitting
BEFT	Back of Elbow to Fingertip	The linear distance between the back of the elbow to the beginning of the middle finger.
SFT	Shoulder Finger Tips Depth	The linear distance between the shoulder to the finger tips when standing
PB	Palm Breadth	Linear distance from the edge of the hand from the small finger to the other side through index
HAB	Hand Breadth	The linear distance from the edge of the hand to the other side through the thumb
WB	Wrist Breadth	The breadth of the wrist
IFH	Index Finger Height	The length of the hand through the index finger
MFH	Middle Finger Height	Maximum length of the hand through the middle finger.
SHF	Seat Height	The linear distance between the siting to floor
PH	Popliteal Height (Sitting)	The linear distance between the back of the knee to the floor
TKH	Top of Knee Height (Sitting)	The linear distance between the top of the knee to the floor when sitting
LPD	Lumbar Popliteal Depth	The linear distance between the lumbar to the back of the knee

**Fig. 2:**
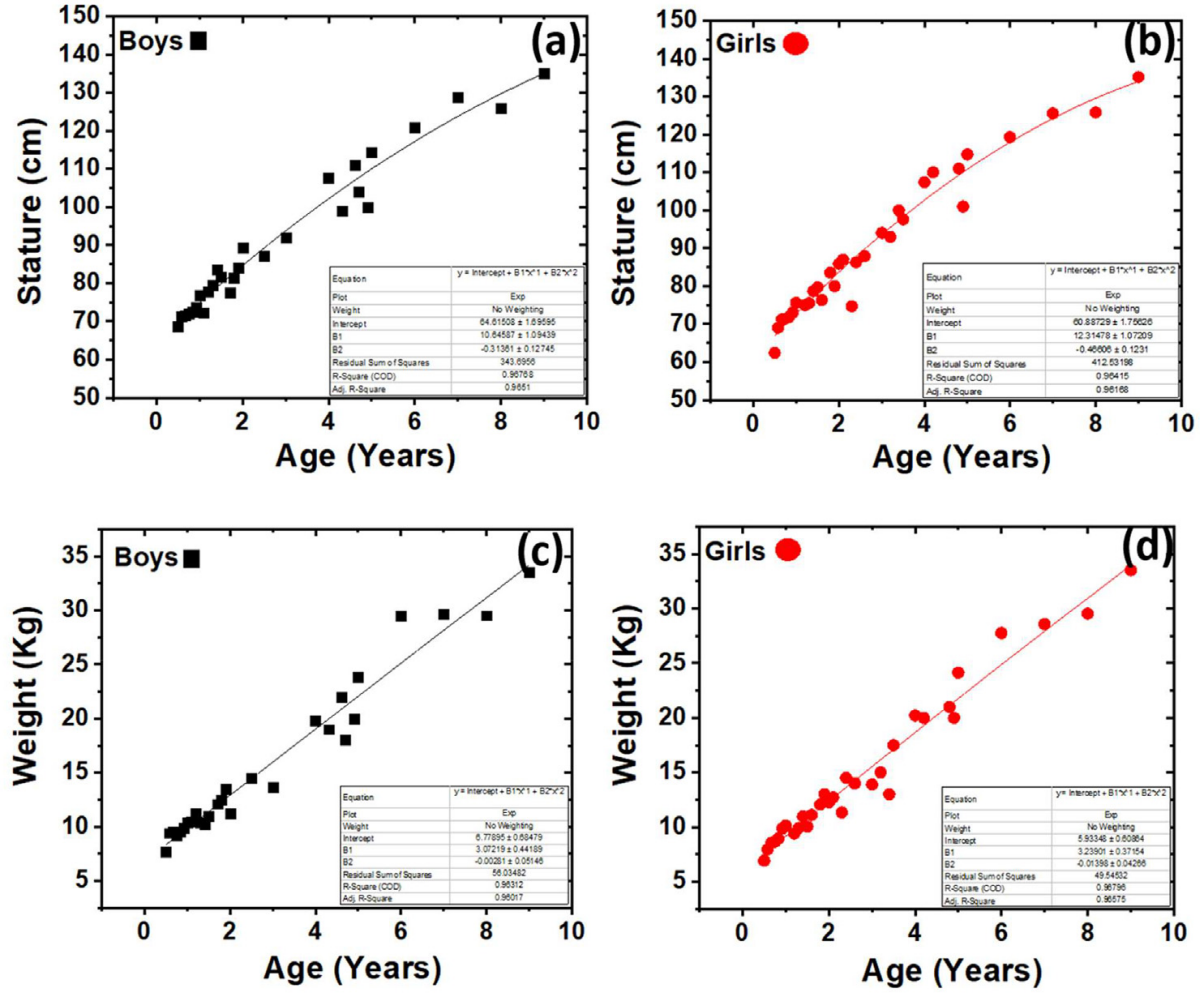
The 50th percentile distribution of the data (a) Stature for boys (b) Stature for girls (c) Weight for boys (d) weight for girls, the inset shows the exponential distribution parameter.

Fig. 2 displays a representative depiction of the distribution at the 50th percentile for Stature and weight over various age intervals for both males and females. The data presents growth charts for children in Jordan, which have significant value in the development of various tools and equipment pertaining to child growth, including clothing, tools, and toys. The data may also be fitted using a statistical tool in order to get the optimal curve and equation that accurately reflect the underlying model. The height and weight measurements for both boys and females were determined to follow an exponential distribution. The data fit may be seen in the inset located on the right side of each image. Nevertheless, there were no noteworthy disparities seen in terms of size and weight between boys and girls.

**Fig. 3:**
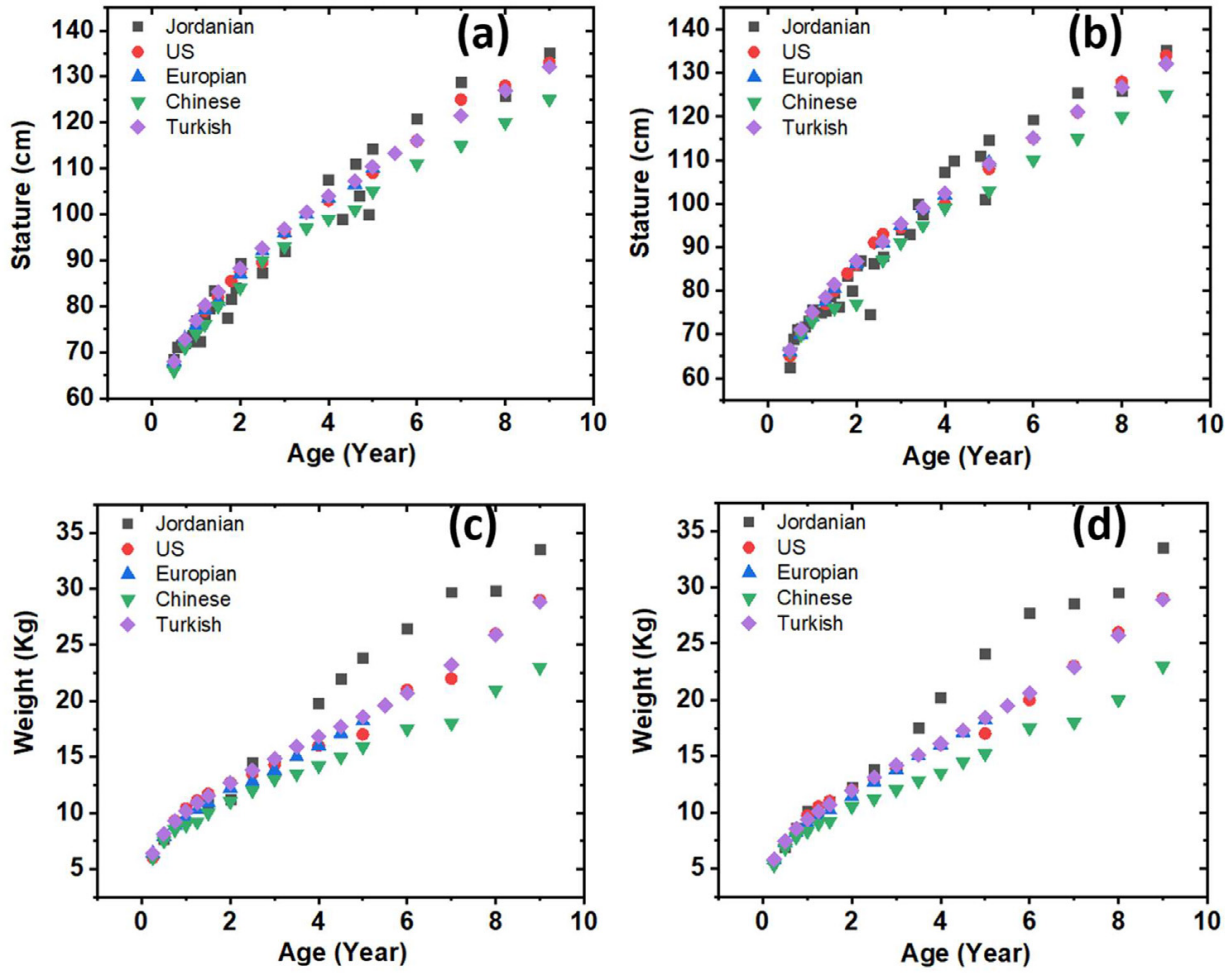
The 50th percentile distribution of the data in comparison with the US, Europe, Chinese, Turkish showing (a) Stature for boys (b) Stature for girls (c) Weight for boys (d) weight for girls.

Fig. 3 illustrates the dispersion of the 50th percentile for stature and weight over various age intervals and genders, in contrast to other countries such the United States, Europe, China, and Turkey [3], [4], [5], [6]. The data shown in Fig. 3(c) and (d) illustrates significant variations in weight among Jordanian children aged four years and older. The observed variations indicate that it is advisable for youngsters in Jordan to own personalized designs pertaining to furniture, tools, and other related items.

## 4 Experimental Design, Materials and Methods

This section provides a comprehensive explanation of the methodological framework, participant demographics, data gathering, and apparatus used in the present study. This research sought the participation of youngsters from various places in Jordan, and a total of 354 juveniles freely took part in this academic endeavor. The participants demonstrated an understanding of the primary goal of the study, which is to provide valuable knowledge that may support the creation of a diverse range of practical goods.

The dataset being analyzed consists of a group of 354 individuals, ranging in age from 6 months to 9 years. The study used anthropometric techniques as the fundamental approach for measuring the physical measurements of youngsters and analyzing specific anatomical features.

According to the data supplied by the department of statistics in Jordan, the children with less than 9 years old are with percentage of 23.75% of the total Jordanian populations (about 10.5 Million). The total children population (N) was calculated to be about 2.5 Million. The sample size was calculated using the following statistics equation:

(1) $$Sample\;Size=\frac{\frac{z^{2}\times p\left(1-p\right)}{e^{2}}}{1+\left(\frac{z^{2}\times p\left(1-p\right)}{e^{2}N}\right)}\;$$

Where *N* is the total population (2.5 million), *e* is the margin of error (0.05 is used), *z* is the z-score (1.65 for 90% confidence level) and *p* is the standard deviation among responses (0.5 is used for worst case) where 90% confidence level and 5% of marginal error were used. The used of the previous equation resulted of sample size of 273 children. More sample size means more accurate results, so that a total number of 354 was adapted and collected from kindergartens, schools and child care centers from different main Jordanian cities (Amman, Zarqa and Ibrid). These cities are considered to be the largest cities in Jordan. The cities have the same environment and the same life style for children. However, the 354 children were selected randomly from these cities. The measurements were taken once for each children and for each anthropometric measure. Different trials mean nothing in this case due to the accuracy and the applied measurement tools. Moreover, dealing with children will not give the measurement team the opportunity to take different measures due to the difficulty in children behavior and stability.

To ensure the accuracy and consistency of these measures, the study used particular anthropometric instruments, including the Lafayette Caliper, Lafayette Chest Depth gauge, and a standard Scale. The selection of these tools was based on their demonstrated precision in obtaining anthropometric data. Additional information on the attributes and technical parameters of these measuring devices may be obtained by referring to Table 1.

## Limitations

None.

## Ethics Statement

This research followed ethical guidelines, ensuring that all subjects gave informed consent. The consent method required a clear explanation of study facts and confirmed participants' voluntary acceptance.

## CRediT authorship contribution statement

**Mumen Rababah:** Data curation, Writing – original draft. **Morad Etier:** Data curation, Writing – review & editing.

## Data Availability

Anthropometric Data Compilation for Children in Jordan: A Comprehensive Dataset (Original data) (Mendeley).
